# BAFF and APRIL immunotherapy following Bacille Calmette-Guérin vaccination enhances protection against pulmonary tuberculosis in mice

**DOI:** 10.3389/fimmu.2025.1551183

**Published:** 2025-02-06

**Authors:** Min Xie, Chen-Yu Tsai, Joshua Woo, Frank Nuritdinov, Melissa Cristaldo, Narineh M. Odjourian, Rosleine Antilus-Sainte, Maureen Dougher, Martin Gengenbacher

**Affiliations:** ^1^ Center for Discovery and Innovation, Hackensack Meridian Health, Nutley, NJ, United States; ^2^ Department of Medical Sciences, Hackensack Meridian School of Medicine, Nutley, NJ, United States

**Keywords:** BCG, unconventional B cells, marginal-zone B cells, immunotherapy, memory T cells, mycobacterium tuberculosis

## Abstract

**Introduction:**

Bacille Calmette-Guérin (BCG), the only tuberculosis vaccine currently in clinical use, provides inadequate long-term protection. Administered at birth, BCG induces broad immune responses against a large number of antigens shared with *Mycobacterium tuberculosis* (Mtb), but protection wanes over time. We have previously shown that unconventional B cell subsets play a role in tuberculosis control.

**Methods:**

High-dimensional flow cytometry and multiplex cytokine analysis were employed to investigate the effects of immunotherapy on BCG-vaccinated mice in an Mtb challenge model.

**Results:**

In this study, we investigate the potential of recombinant cytokines targeting B cells – B-cell activating factor (BAFF) and A proliferation-inducing ligand (APRIL) – to modulate BCG immunity and improve protection in mice. Both cytokines play overlapping roles in B cell development and peripheral survival. Following subcutaneous BCG vaccination, immunotherapy with BAFF or APRIL resulted in an increased frequency of unconventional B cells potentially transitioning into antibody-producing plasma cells. Concurrently, we observed an increased frequency of central memory T cells, a subset critical for protective immunity. Changes in cellular immune responses were accompanied by reduced pro-inflammatory cytokine profiles and a contraction of the leukocyte population in lungs. Importantly, mice receiving BCG vaccination followed by BAFF or APRIL immunotherapy exhibited superior long-term protection against pulmonary tuberculosis relative to controls that received only BCG.

**Conclusion:**

In summary, our findings demonstrate that combining BCG vaccination with B cell targeted immunomodulatory therapies can improve long-term protection against pulmonary tuberculosis, highlighting the continued relevance and underutilized potential of BCG as a vaccine platform.

## Introduction

Tuberculosis (TB) continues to be one of the leading causes of death from a single infectious agent, accounting worldwide for 10.8 million new cases and 1.25 million deaths in 2023 (1). While effective drug regiments are available, treatment is lengthy, costly and faces tremendous challenges through the rise of drug-resistant Mtb requiring the use of more toxic and less effective drugs (2). The only TB vaccine currently in clinical use is Bacillus Calmette-Guérin (BCG), which was derived from the bovine-TB pathogen *Mycobacterium bovis* by serial passaging that led to attenuation (3). BCG given intradermally at birth, is very safe in the human population except in immunocompromised individuals that may develop a TB-like disseminated diseases known as BCGosis (4). BCG is highly effective in preventing disseminated TB and tuberculous meningitis in children. However, its protection against pulmonary TB in adolescents and adults – which account for over 90% of TB cases – is unreliable underscoring the urgent need for a robust vaccine development pipeline and novel immunization strategies (5, 6). Currently, more than 17 TB vaccine candidates are in clinical development including live vaccines, inactivated whole-cell preparations, subunit vaccines, viral vectors and mRNA vaccines aiming to overcome the limitations of BCG (7).

The induction of memory T cells is critical for effective TB vaccines (8). In particular, CD4 T cells with T helper 1 (Th1) phenotype in response to subcutaneous BCG vaccination are important for protection in the murine challenge model (9, 10). Polyfunctional CD4 T cells producing pro-inflammatory cytokines interferon(IFN)-γ, interleukin(IL)-2 and tumor necrosis factor(TNF)-α have been suggested as correlate of protection but other functional attributes including additional effector functions, memory T cell phenotypes, tissue homing potential, and long-term survival capacity may be equally or more important to promote protection (11). Several functional subsets of memory T cells have been described: (i) central memory T cells (T_CM_) recirculate between blood and lymph nodes and maintaining long-term immunological memory, (ii) effector memory T cells (T_EM_) transitioning between blood and peripheral tissues where they primarily reside to defend against pathogens, and (iii) tissue resident memory T cells (T_RM_) are permanently located in peripheral tissues to deploy their rapid recall capabilities against intruders (12). Although their full protective potential in animal models and humans is not yet completely defined, targeting memory T cells is of high interest for TB vaccine development (13, 14).

We recently demonstrated that TB infection in mice leads to the expansion of marginal-zone B (MZB) cells, a subset of unconventional B cells, in the spleen and – unexpectedly – in the lungs (15). MZB cells were activated, had a memory phenotype and contributed to systemic Mtb control by shaping the cytokine pattern and cell-mediated immunity (15). These findings motivated us to determine whether BCG also elicits unconventional B cells that can be harnessed for TB vaccine development. The strength of BCG lays in its capacity to elicit immune responses to a broad range of mycobacterial antigens that are shared with Mtb (16). We hypothesized that BCG vaccination leads to changes in unconventional B cell subsets that could be enhanced by immunotherapy with the cytokines B-cell activating factor (BAFF) and A proliferation-inducing ligand (APRIL). Both cytokines have a functional overlap and play major roles in controlling B cell maturation, differentiation and peripheral survival (17). Since constitutive overexpression of BAFF or APRIL is associated with autoimmune conditions (18, 19) we opted for transient administration of the recombinant cytokines after BCG prime vaccination to test our hypothesis.

## Materials and methods

### Bacterial strains and growth conditions

Mtb H37Rv (ATCC #27294) and BCG Danish 1331 (ATCC #35733) were grown in Middlebrook 7H9 broth supplemented with 10% (vol/vol) Middlebrook ADC growth supplement (Sigma-Aldrich), 0.2% glycerol, and 0.05% Tween 80. Cultures were grown at 37°C in 1 L roller bottles (Corning, 490 cm^2^ surface area) rotated at 2 rpm until reaching the mid-log phase.

### Vaccine preparation

For vaccine stock preparations, log-phase BCG were harvested, resuspended in PBS with 10% glycerol to a final optical density (OD_600_) of 3, and stored at -80°C. The viability of vaccine stocks was verified in regular intervals by plating samples on Middlebrook agar. Prior to vaccination, frozen vials were thawed, cells were pelleted and resuspended in PBS containing 0.05% tyloxapol for administration.

### Animal experimentation

Ten-week-old female, specific pathogen-free BALB/c mice were purchased from Charles River Laboratory and housed in groups of five in individually ventilated cages in the Animal Biosafety Level-3 laboratory at the Center for Discovery and Innovation, Hackensack Meridian Health. Mice were vaccinated subcutaneously at the lower back with BCG (10^6^ colony-forming unit (CFU)/mouse). The vaccine dose was verified by plating samples on Middlebrook agar. From 30 to 60 days post-vaccination, groups of mice were administered weekly intraperitoneal injections of recombinant BAFF (R&D Systems, #8876-CF, 10 μg/mouse) or APRIL (R&D Systems, #7907-CF, 10 μg/mouse). At 90 days post-vaccination, mice were infected with 100-200 CFU of aerosolized Mtb H37Rv using a full-body inhalation exposure system (Glas-Col). Mice were euthanized at designated time points for subsequent analyses. Tissues, including lungs, spleen, and/or inguinal lymph nodes, were collected to determine protective efficacy, perform multiplex cytokine/chemokine assays, and measure cellular immune responses.

### Protective efficacy in mice

At designated time points post-infection, lungs and spleen were aseptically removed and homogenized in 1 mL PBS containing 0.05% Tween 80 (PBST) in gentleMACS M Tubes using the gentleMACS Dissociator (Miltenyi Biotec) for CFU enumeration. Organ homogenates were serially diluted in PBST. Diluted samples were plated on Middlebrook 7H11 agar (Sigma-Aldrich) supplemented with 10% (vol/vol) Middlebrook OADC enrichment (Becton, Dickinson) and 0.5% glycerol. Plates were incubated at 37°C for 3 to 4 weeks before CFU enumeration.

### Multiplex cytokine/chemokine assay

At designated time points post-vaccination and post-infection, tissues were aseptically removed and homogenized in PBST (1 mL for lungs and spleen, 0.2 mL for inguinal lymph nodes) in gentleMACS M Tubes using the gentleMACS dissociator. Cytokine and chemokine levels in the tissue homogenates were quantified using the Milliplex Mouse Cytokine/Chemokine Magnetic Bead Panel – Premixed 32 Plex – Immunology Multiplex Assay (Millipore), following the manufacturer’s protocol. The assay plates were analyzed using the FLEXMAP 3D system (Millipore) according to the manufacturer’s instructions. Raw data were processed and analyzed using Belysa software (version 1.2.1, Millipore).

### Flow cytometry analysis

At designated time points post-vaccination, lungs and spleen were aseptically removed and single-cell suspensions of each organ were prepared as previously described (15). Organ cell suspensions were normalized, seeded, stained and subjected to flow cytometry analysis (BD FACSymphony A3, BD Biosciences) as previously described (15). Antibodies and reagents used in the analyses include (i) B cell panel: CD45-Alexa Fluor700 (30-F11) (Biolegend), CD19-BV785 (6D5) (Biolegend), B220-BUV395 (RA3-6B2) (BD Bioscience), CD93-PE.Cy7 (AA4.1) (Biolegend), CD21/35-FITC (7E9) (Biolegend), CD23-PE (B3B4) (Biolegend), CD1d-BV421 (1B1) (BD Bioscience), CD138-BV711 (281-2) (BD Bioscience), CD69-BV605 (H1.2F3) (Biolegend), CD86-APC (GL-1) (Biolegend), CD43-BV480 (S7) (BD Bioscience), CD11c-BB700 (HL3) (BD Bioscience) and the dump channel: CD3-APC-eFluor780 (17A2) (eBioscience), CD355-APC-eFluor780 (29A1.4) (eBioscience), LIVE/DEAD™ Fixable Near-IR Dead Cell Stain Kit (Thermo Fisher Scientific); (ii) T cell panel: CD45-Alexa Fluor 700 (30-F11) (Biolegend), CD3-BV605 (17-A2) (Biolegend), CD4-BUV395 (GK1.5) (BD Bioscience), CD8a-BB700 (53-6.7) (BD Bioscience), CD69-PE (H1.2F3) (Biolegend), CD103-BV421 (2E7) (Biolegend), gdTCR-FITC (GL3) (Biolegend), NK335-PE.Cy7 (29A1.4) (Biolegend), B220-BUV496 (RA3-6B2) (BD Bioscience), PD-1-BV785 (29F.1A12) (Biolegend), CXCR3-APC (CXCR3-173) (BD Bioscience), CD62L-BV480 (MEL-14) (BD Bioscience), CD44-BUV805 (IM7) (BD Bioscience), LIVE/DEAD™ Fixable Near-IR Dead Cell Stain Kit (Thermo Fisher Scientific). Absolute cell counts were determined using counting beads (CountBright, Thermo Fisher Scientific). Flow cytometry data were analyzed by FlowJo software (version 10.10.0, BD Biosciences). The gating strategies are depicted in **Supplementary Figure 1A** and **2A**.

### Statistical analyses

GraphPad Prism software (version 10.4.0, GraphPad) was used for data analysis. Results were represented as mean ± standard deviation (SD). One-way ANOVA with Dunnett’s multiple-comparison *post hoc* test was used to determine the statistical differences between groups. A *p*-value <0.05 was considered statistically significant.

## Results

### Splenic MZB cells expand in response to subcutaneous BCG vaccination

**Figure 1:**
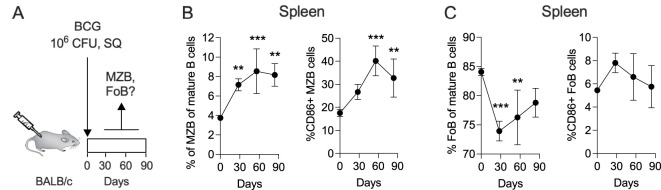
The frequency of splenic MZB cells increases in response to subcutaneous BCG vaccination. **(A)** Ten-weeks-old BALB/c mice were subcutaneously vaccinated with 10^6^ CFU of BCG Danish at the lower back. At 30-, 60- and 90-days post vaccination, groups of 4 mice were euthanized to prepare single cell suspensions for flow cytometry analysis (**Supplementary Figure 1A**). The flow cytometry data represent total B cell populations. Frequencies of MZB cells **(B)**, FoB cells **(C)** and their CD86+ subsets are shown, respectively. Mean and SD are shown. Statistical analysis was performed by one-way ANOVA and multiple comparisons by Dunnett’s posttest using the pre-vaccination time point (=day 0) as a reference. One dataset out of two biological replicates is shown. **p<0.01; ***p<0.001.

MZB cells (B220+ CD93+ CD1d^hi^ CD23^lo^) are an important component of the immune system. Due to their strategic location in the spleen, MZB cells quickly encounter circulating antigens, blood-borne viruses and encapsulated bacteria to produce antibodies, essentially acting as innate-like B cells that play a primary role in early immune responses (20). In mice, splenic MZB cells expand during both the acute and chronic phases of TB infection (15). Although BCG lacks certain virulent regions found in Mtb, their genomes are 99.9% identical (21, 22). To determine whether BCG vaccination induces a similar expansion of MZB as observed during Mtb infection, we vaccinated mice subcutaneously with BCG and measured the frequency of MZB cells over time by flow cytometry (**Figure 1A** and **Supplementary Figure 1A**). We also analyzed follicular B (FoB; B220+ CD93+ CD1d^mi^ CD23^hi^) cells, another subset of unconventional B cells involved in T cell-dependent antibody responses, T cell-independent responses to blood-borne pathogens, and generation of memory B cells (23). Following BCG vaccination, the frequency of splenic MZB cells among all mature B cells increased significantly, rising from approximately 4% at baseline to 8% at day 30, and plateauing by day 60. Similarly, the proportion of activated CD86+ MZB cells doubled within 60 days post-immunization (**Figure 1B**). In contrast, the frequency of FoB cells declined sharply until day 30 but recovered progressively thereafter, with no significant changes in the proportion of activated CD86+ FoB cells (**Figure 1C**). In principle, these results closely mirror the behavior of MZB and FoB cells in the spleen of Mtb-infected mice (23), despite differences in the administration route (subcutaneous for BCG vs. aerosol for Mtb) and inoculum size (10^6^ CFU for BCG vs. 10^2^ CFU for Mtb). We conclude that BCG-derived antigens lead to a prolonged expansion and activation of splenic MZB cells while the frequency of FoB cells only temporarily dropped.

### Immunotherapy with BAFF or APRIL following BCG vaccination modulates unconventional B cells and enhances central memory T cells

**Figure 2:**
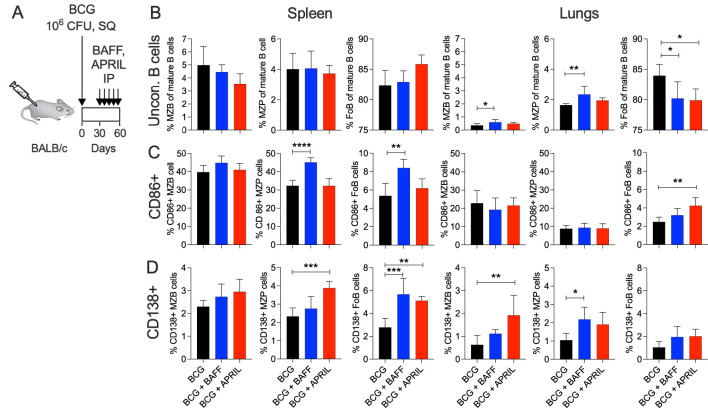
BCG vaccination followed by B cell-targeting immunotherapy increased the frequency of unconventional B cells to transition into plasma cells. **(A)** Ten-weeks-old BALB/c mice were subcutaneously vaccinated with 10^6^ CFU of BCG Danish at the lower back. From day 30 to 60 post vaccination cohorts of BCG-vaccinated mice received weekly intraperitoneal injections with 10 μg BAFF or APRIL. Five mice per group were euthanized at day 60 (48 h after the last cytokine injection) to prepare single cell suspensions of organs for flow cytometry analysis of total B cell populations (**Supplementary Figure 1A**). **(B)** Frequencies of MZB, MZP and FoB cells in the spleen and in lungs. **(C)** Frequencies of CD86+ MZB, MZP and FoB cells in the spleen and in lungs. **(D)** Frequencies of CD138+ MZB, MZP and FoB cells in the spleen and in lungs. Mean and SD are shown. Statistical analysis was performed by one-way ANOVA and multiple comparisons by Dunnett’s posttest using the BCG-vaccinated group as a reference. *p<0.05; **p<0.01; ***p<0.001; ****p<0.0001.

To investigate whether targeting MZB cells can modulate BCG-induced B cell and T cell immunity, we administered the B-cell cytokines BAFF or APRIL at the time of peak MZB cell frequency. Mice received five weekly injections of BAFF or APRIL (10 μg/mouse) from day 30 to day 60 post BCG vaccination. B and T cell responses were analyzed upon completion of the immunotherapy (**Figure 2A**). Single cell suspensions from the spleen and lungs of BCG, BCG+BAFF and BCG+APRIL cohorts were subjected to flow cytometry, using gating strategies depicted in **Supplementary Figure 1A** and **2A**. Immunotherapy with BAFF or APRIL significantly altered pulmonary unconventional B lymphocytes. The frequencies of pulmonary MZB (B220+ CD93+ CD1d^hi^ CD23^lo^) and their precursor (MZP; B220+ CD93+ CD1d^hi^ CD23^hi^) cells increased significantly in the BCG+BAFF cohort and showed a trend toward increase in the BCG+APRIL cohort compared to the BCG-only cohort (**Figure 2B**, Lungs). The frequency of pulmonary FoB cells (B220+ CD93+ CD1d^mi^ CD23^hi^) decreased significantly in both cytokine-treated groups compared to BCG-only controls (**Figure 2B**, Lungs), suggesting a shift in the pool of mature B lymphocytes from FoB cells to other subsets, including MZB and MZP cells. Interestingly, the frequencies of unconventional B lymphocytes in the spleen remained unaffected by cytokine therapy (**Figure 2B**, Spleen). Absolute numbers of MZB, MZP and FoB in both the spleen and lungs were unchanged by cytokine administration (**Supplementary Figure 1B**). However, BAFF therapy increased the frequency of activated (CD86+) MZB, MZP and FoB cells in the spleen, though it is not statistically significant for MZB cells (**Figure 2C**, Spleen). In the lungs, APRIL therapy increased the proportion of activated (CD86+) FoB cells (**Figure 2C**, Lungs). Notably, BAFF and APRIL immunotherapy enhanced the frequency of unconventional B cell subsets expressing the plasma cell marker CD138 in both spleen and lungs (**Figure 2D**), suggesting a potential transition to antibody-producing plasma cells.

**Figure 3:**
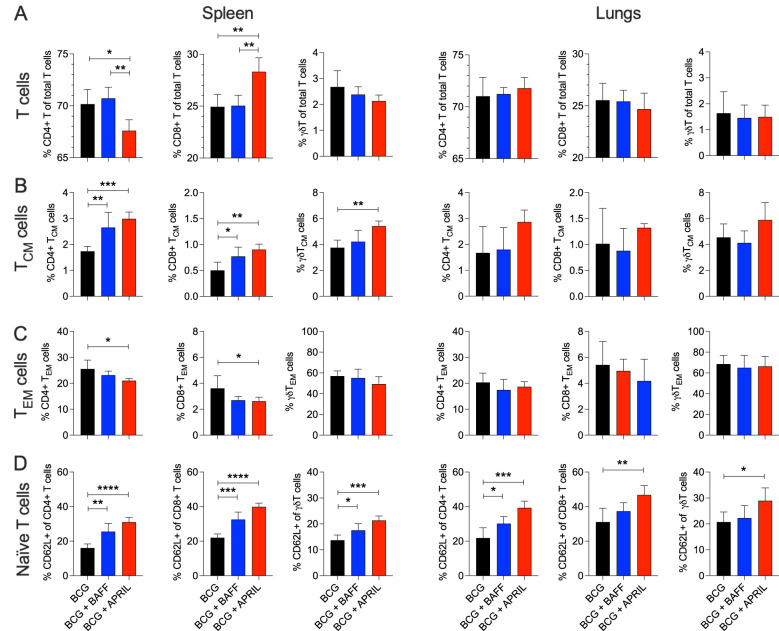
BCG vaccination followed by B cell-targeting immunotherapy increased the frequency of central memory T cells in the spleen. Ten-weeks-old BALB/c mice were subcutaneously vaccinated with 10^6^ CFU of BCG Danish at the lower back prior to undergoing BAFF or APRIL treatment (**Figure 2A**). Five mice per group were euthanized at day 60 (48 h after the last cytokine injection) to prepare single cell suspensions of organs for flow cytometry analysis of total T cell populations (**Supplementary Figure 2A**). **(A)** Frequencies of total CD4, CD8, and γδT cells in the spleen and in lungs. **(B)** Frequencies of central memory (CM) CD4, CD8, and γδT cells in the spleen and in lungs. **(C)** Frequencies of effector memory (EM) CD4, CD8, and γδT cells in the spleen and in lungs. **(D)** Frequencies of naïve CD4, CD8, and γδT cells in the spleen and in lungs. Mean and SD are shown. Statistical analysis was performed by one-way ANOVA and multiple comparisons by Dunnett’s posttest using the BCG-vaccinated group as a reference. *p<0.05; **p<0.01; ***p<0.001; ****p<0.0001.

Next, we analyzed the impact of cytokine therapy on T cell subsets and their memory phenotypes, which are critical for protective immunity against TB. In the spleen, APRIL therapy significantly altered the ratio of CD4 and CD8 T cells (**Figure 3A**, Spleen). Both BAFF and APRIL therapy significantly increased the frequencies of splenic central memory CD4, CD8, and γδT cells (**Figure 3B**, Spleen). Effector memory CD4 and CD8 T cell frequencies were reduced by cytokine therapy, with significant reductions observed in the APRIL-treated group (**Figure 3C**, Spleen). No significant changes in pulmonary T cell subsets or their memory phenotypes were detected among the study groups (**Figures 3A-C** and **Supplementary Figure 2B**). In contrast, BAFF and APRIL immunotherapy resulted an increase in the frequencies of naïve CD4, CD8, and/or γδT cells in the spleen and the lungs (**Figure 3D**), suggesting a decreased proportion of T cells had undergone differentiation. The absolute numbers of total CD4, CD8, and γδT cells in the spleen and lungs remained unchanged by cytokine treatment (**Supplementary Figure 2C**). Taken together, immunotherapy with BAFF or APRIL moderately increased the frequencies of MZB and MZP cells in lungs and enhanced the proportions of activated MZP and central memory T cells, including CD4, CD8, and γδ subsets, in the spleen. These changes highlight the potential of B cell-targeted cytokine therapies to improve humoral and cellular immunity critical for protective immunity against TB.

### Immunotherapy with BAFF or APRIL following BCG-vaccination generates a less inflammatory environment in draining lymph nodes, spleen and lungs

**Figure 4:**
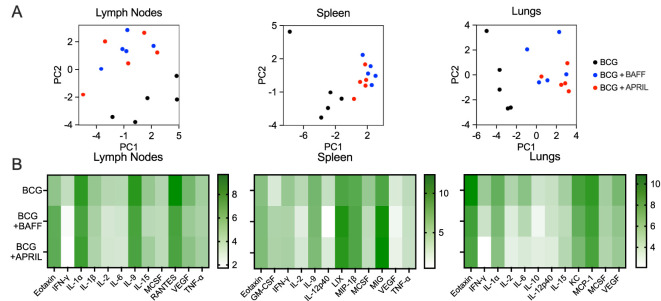
BCG vaccination followed by B cell-targeting cytokine therapy shifted organ cytokine and chemokine patterns. Ten-weeks-old BALB/c mice were subcutaneously vaccinated with 10^6^ CFU of BCG Danish at the lower back prior to undergoing BAFF or APRIL treatment (**Figure 2A**). Five mice per group were euthanized on day 60 (48 h after the last cytokine injection) for cytokine/chemokine quantification in organ homogenates **(A)** Principal component analysis of cytokine/chemokine protein levels in the inguinal lymph nodes, spleen and lungs. Each data point represents the cytokine profile of an individual animal. **(B)** Heatmaps showing the grouped mean cytokine levels (natural log-transformed concentrations, In[pg/mL]) in the inguinal lymph nodes, spleen and lungs. Each row represents the mean cytokine profile for the designated treatment group and each column represents an individual cytokine.

To probe for changes in the immune environment related to BAFF or APRIL therapy after BCG vaccination, we quantified the levels of 32 cytokines and chemokines in homogenates of draining lymph nodes, spleen and lungs as BCG disseminates to these organs in the weeks following subcutaneous immunization (24). BCG induces profound changes to cytokine and chemokine patterns in mice and in infants and vary based on factors such as the Mtb isolate, vaccine dose and mouse strain (25), as well as human population factors (26), respectively. We first performed a principal component analysis (PCA) to visualize the impact of BAFF or APRIL administration in the context of BCG vaccination. Cytokine profiles of draining lymph nodes, spleen and lungs from mice that received BCG-only showed distinct clustering, clearly separated from those of BCG+BAFF and BCG+APRIL groups (**Figure 4A**, black dots). In contrast, data points from the BCG+BAFF and BCG+APRIL groups were intermixed across all three tested organs (**Figure 4A**, blue and red dots), indicating similar cytokine profiles between these two treatment groups. This finding is consistent with the reported overlapping biological functions and receptors shared by BAFF and APRIL (17). Heatmaps of the 12 most differentially expressed cytokines and chemokines in each tested organ confirm the distinct pattern of BCG-only cohort compared to the other groups (**Figure 4B**). Overall, cytokines and chemokines concentrations were lower in the BCG+BAFF and BCG+APRIL groups relative to BCG controls (**Figure 4B** and **Supplementary Table 1**). Exceptions include: eotaxin (chemoattractant of eosinophiles) (27) increased in the lymph nodes and spleen, lipopolysaccharide-induced CXC chemokine (LIX; cell migration and activation of neutrophiles) (28), granulocyte-macrophage colony-stimulating factor (GM-CSF; development, differentiation and activation of macrophages and granulocytes) (29) and monokine induced by IFN-γ (MIG; chemokine recruiting activated T cells to infection site) (30) increased in the spleen. Notably, cytokines involved in both innate and adaptive immunity and playing a key role in T cell memory, including IFN-γ and IL-2 (31), were remarkably reduced in BCG-BAFF and BCG-APRIL groups by the end of immunotherapy (day 60) (**Figure 4B**). Overall, immunotherapy with BAFF or APRIL reduced the concentrations of many pro-inflammatory cytokines and chemokines, resulting in a less inflammatory environment. This effect was most pronounced in the lungs, suggesting a potential organ specific role of cytokine modulation in shaping the immune response following BCG vaccination.

### Immunotherapy with BAFF or APRIL following BCG-vaccination provided superior protection against pulmonary TB

**Figure 5:**
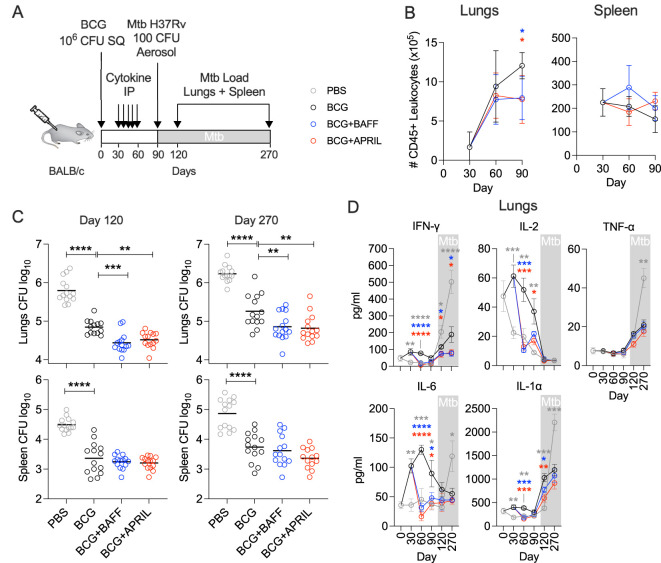
BCG vaccination followed by BAFF or APRIL administration improves protection from Mtb challenge. **(A)** Ten-weeks-old BALB/c mice were subcutaneously vaccinated with 10^6^ CFU of BCG Danish or with PBS. From day 30 to 60 post vaccination cohorts of BCG-vaccinated mice received weekly intraperitoneal injections with 10 μg BAFF or APRIL in PBS. Animals receiving PBS only served as controls. At 90 days post immunization, all mice were challenged with 100-200 CFU Mtb H37Rv delivered by aerosol. The inoculum was verified 1 day after infection using 4 mice. **(B)** Total lymphocytes in lungs and spleen during the vaccination phase. **(C)** At day 120 and 270 post vaccination corresponding to 30- and 180-days post Mtb challenge, groups of animals were euthanized to determine the Mtb load in lungs and spleen by plating organ homogenates on agar. Mean and individual data points of two pooled biological replicates are shown. **(D)** At designated time points lung homogenates of 5-7 mice were used to quantify cytokines. Statistical analysis was performed by one-way ANOVA and multiple comparisons by Dunnett’s posttest using the BCG-vaccinated group as a reference. *p<0.05; **p<0.01; ***p<0.001; ****p<0.0001.

We previously demonstrated that BAFF or APRIL therapy alter the immunological trajectories of BCG vaccination in mice, including changes in the unconventional B cell subsets, T cell subsets and memory phenotype, and reduced levels of pro-inflammatory cytokines and chemokines. To evaluate whether the changes translate into improved protection, we employed a previously developed prophylactic vaccine efficacy model (24, 32, 33). Mice immunized with BCG and injected with recombinant BAFF or APRIL (weekly, from day 30 to day 60 post-BCG) were challenged with a low dose of aerosolized Mtb at day 90 post-BCG. Protection was assessed by determining the Mtb burden in the lungs and spleen at days 120 and 270 post-BCG (**Figure 5A**). The earlier observed reduction in pro-inflammatory cytokines (**Figure 4**) prompted us to quantify total number of leukocytes during the vaccination phase (day 0 to day 90) using the pan-leukocyte marker CD45 (15) (**Figure 5A**). In the lungs, the number of CD45+ immune cells steadily increased until day 90 in the BCG-only group, whereas leukocyte counts remained stable after day 60 in the BCG+BAFF and BCG+APRIL groups (**Figure 5B**, Lungs). In the spleen, total CD45+ immune cell counts remained comparable across all groups during the vaccination phase (**Figure 5B**, Spleen). Consistent with previous reports (24, 32, 33), BCG vaccination alone elicited a robust protection, evident by ~1 log reduction in the pathogen load of lungs and spleen relative to PBS controls throughout the infection phase (**Figure 5C**). Mice receiving BCG+BAFF or BCG+APRIL exhibited superior pulmonary protection, with Mtb burden in the lungs reduced by ~1.5 logs and ~0.5 logs compared to the PBS and BCG-only controls, respectively (**Figure 5C**). However, BAFF or APRIL immunotherapy did not reduce the pathogen load in the spleen, suggesting that the increased frequencies of splenic central memory T cells (**Figure 3B**) did not directly translate into improved protection in this organ (**Figure 5C**). To gain insights into the superior pulmonary protection observed in BCG+BAFF and BCG+APRIL groups, we measured lung levels of Th1 cytokines (IFN-γ, IL-2, TNF-α) and other pro-inflammatory cytokines (IL-6, IL-1α) during the vaccination phase (day 0 to day 90) and the Mtb challenge phase (day 90 to day 270) (**Figure 5A**). Overall, the PBS controls showed only minor changes in cytokine levels during the vaccination phase and the highest cytokine levels during TB infection (**Figure 5D**). Consistent with our single-point cytokine/chemokine analysis (**Figure 4**), BAFF or APRIL immunotherapy led to a sharp decline in IFN-γ, IL-2, IL-6 and IL-1α at day 60 while TNF-α levels remain unaffected relative to the BCG-only controls (**Figure 5D**). These changes were transient, and cytokine levels rebounded until the end of our study, except for IFN-γ, which remained higher in the cohort receiving only BCG throughout TB infection (**Figure 5D**). Notably, pulmonary IFN-γ levels correlated with the corresponding Mtb burden across all groups (**Figure 5C, D**).

In summary, immunotherapy with BAFF or APRIL following BCG vaccination reduced inflammatory cytokine levels and the number of immune cells in the lungs during the immunization phase. These changes were associated with enhanced protection against pulmonary TB, highlighting the potential of cytokine-based immunomodulation to improve BCG efficacy.

## Discussion

BCG is a live vaccine with a genome that is 99.9% identical to that Mtb (21, 22). Its exceptional immunogenicity including a large number of antigens shared with Mtb and its ability to persist within the host have sparked significant interest in developing BCG as a versatile vaccine platform (34–36). To date, much of TB vaccine development has focused on enhancing T cell-mediated immunity, while strategies targeting B cells remain underexplored. Unconventional B cells, which we have recently shown to play a key role in controlling TB infection (15), represent an emerging area of interest in TB immunity. In this study, we uniquely emphasized the role of the B cell compartment to leverage the strength of BCG. To our knowledge, this is the first study to demonstrate that modulating immunity through specific B-cell cytokines can significantly enhance the efficacy of BCG. These findings broaden the traditional view of TB vaccine research as being solely T cell-dependent and highlight the potential of unconventional approaches in TB vaccine development.

In our study, we first demonstrated that subcutaneous BCG vaccination increased the frequency of splenic MZB cells (**Figure 1**), a response comparable to that seen during Mtb infection (15). For immunotherapy, we selected the TNF-like cytokines BAFF and APRIL, which are known to influence B cell maturation, peripheral survival, and immunoglobulin class switching, processes with multiple potential clinical implications (37–39). BAFF and APRIL, along with their receptors BAFF-R, TACI and BCMA, share overlapping biological functions. While BCMA expression is restricted to B cells, BAFF-R and TACI are also expressed on activated T cells (38, 40), allowing BAFF and APRIL to interact with both B and T cells. Consistently, we observed changes in both the B and the T cell compartments in BCG-vaccinated mice treated with BAFF or APRIL (**Figure 2** and **3**). Immunotherapy enhanced the frequencies of MZB cells and their precursors in the lungs, as well as unconventional B cells expressing the plasma cell marker CD138+ in both the lungs and spleen (**Figure 2B, D**). The latter may reflect a potential transition to antibody-producing plasma cells. It is important to note that continuous overexpression of BAFF has been associated with the development of autoantibodies and autoimmune conditions (41) and elevated levels of BAFF and APRIL are involved in the pathogenesis of Common Variable Immunodeficiency (42). To mitigate these risks, we limited cytokine-based immunotherapy for only 4 weeks (**Figure 2A**), administered during the period of peak MZB cell frequency (**Figure 1B**). Whether BAFF or APRIL treatment, in combination with BCG vaccination, expands the pool of plasma cells capable of producing mycobacteria-specific antibodies remains an important area of future investigations.

Our strategy combining BCG vaccination and cytokine immunotherapy also led to an increase in the frequency of T_CM_ cells in the spleen (**Figure 3**). T_CM_ cells primarily residing in lymphatic organs, can rapidly proliferate and differentiate into effector cells upon antigen re-encounter, thereby providing long-term protection (13, 43). Their ability to circulate through the blood (44, 45) may have contributed to the superior long-term protection against pulmonary TB observed in the BCG+BAFF and BCG+APRIL groups (**Figure 5C**). Other factors contributing to enhanced organ-specific protection may include increased frequencies of MZB and MZP cells in the lungs (**Figure 2B**), reduced pulmonary cytokine and chemokine levels following BAFF or APRIL therapy at day 60 (**Figure 4**), and decreased leukocyte numbers at day 90 (**Figure 5B**). The cytokine response to BCG is complex, varying with recipient’s age, BCG strain used, and other factors, and no clear correlation between cytokine levels and protection has been established (46). A more detailed analysis of pro-inflammatory cytokines including IFN-γ, IL-2, IL-6, and IL-1α revealed that the observed cytokine downshift at day 60 was transient (**Figure 5D**). Interestingly, IFN-γ levels remained elevated in the lungs of mice receiving only BCG compared to BCG+BAFF and BCG+APRIL groups (**Figure 5D**). Current knowledge suggests that IFN-γ correlates with immunogenicity (46), implying that the superior protection observed in the BCG+BAFF and BCG+APRIL groups likely involves an IFN-γ-independent mechanism and potentially involves humoral immunity. The increased frequency of unconventional B cells expressing CD138+ in our studies (**Figure 2D**) supports this possibility. However, the role of mycobacteria-specific antibodies in the immune response to BCG vaccination and during Mtb infection remains a topic of ongoing debate, with findings in humans and animal models yielding inconsistent results (47).

In conclusion, combining BCG vaccination with BAFF or APRIL cytokine therapy modulates both B and T cell immunity, providing improved and long-lasting protection against pulmonary TB. By strategically targeting the B cell compartment, this study reveals a novel approach of vaccine efficacy that may complement traditional T cell-focused approaches. Moreover, immunotherapy directed at B cells could be explored in the context of BCG as heterologous vaccine platform. By advancing our understanding of the immune response induced by BCG, this work underscores the need to fully explore the potential of BCG and offers a promising direction for future TB vaccine development.

## Data Availability

The original contributions presented in the study are included in the article/**Supplementary Material**. Further inquiries can be directed to the corresponding author.

## References

[1] WHO. Global Tuberculosis Report 2024. Geneva: World Health Organization (2024).

[2] Dobbs TE Webb RM. Chemotherapy of tuberculosis. Microbiol Spectr. (2017) 5:1–16. doi: 10.1128/microbiolspec.TNMI7-0040-2017 PMC11687481 28387179

[3] Kaufmann SHE. Vaccine Development against Tuberculosis over the Last 140 Years: Failure as Part of Success. Front Microbiol. (2021) 12:750124. doi: 10.3389/fmicb.2021.750124 34691001 PMC8526900

[4] Talbot EA Perkins MD Silva SF Frothingham R. Disseminated bacille calmette-guerin disease after vaccination: case report and review. Clin Infect Dis. (1997) 24:1139–46. doi: 10.1086/513642 9195072

[5] Lange C Aaby P Behr MA Donald PR Kaufmann SHE Netea MG. 100 years of mycobacterium bovis bacille calmette-guerin. Lancet Infect Dis. (2022) 22:e2–e12. doi: 10.1016/S1473-3099(21)00403-5 34506734 PMC11967564

[6] Kaufmann SHE. Vaccination against tuberculosis: revamping bcg by molecular genetics guided by immunology. Front Immunol. (2020) 11:316. doi: 10.3389/fimmu.2020.00316 32174919 PMC7056705

[7] da Costa C Benn CS Nyirenda T Mpabalwani E Grewal HMS Ahmed R. Perspectives on development and advancement of new tuberculosis vaccines. Int J Infect Dis. (2024) 141S:106987. doi: 10.1016/j.ijid.2024.106987 38417616

[8] Ottenhoff TH Kaufmann SH. Vaccines against tuberculosis: where are we and where do we need to go? PloS Pathog. (2012) 8:e1002607. doi: 10.1371/journal.ppat.1002607 22589713 PMC3349743

[9] Cowley SC Elkins KL. Cd4+ T cells mediate ifn-gamma-independent control of mycobacterium tuberculosis infection both *in vitro* and *in vivo* . J Immunol. (2003) 171:4689–99. doi: 10.4049/jimmunol.171.9.4689 14568944

[10] Andersen P Smedegaard B. Cd4(+) T-cell subsets that mediate immunological memory to mycobacterium tuberculosis infection in mice. Infect Immun. (2000) 68:621–9. doi: 10.1128/IAI.68.2.621-629.2000 PMC97184 10639425

[11] Lewinsohn DA Lewinsohn DM Scriba TJ. Polyfunctional cd4(+) T cells as targets for tuberculosis vaccination. Front Immunol. (2017) 8:1262. doi: 10.3389/fimmu.2017.01262 29051764 PMC5633696

[12] Kirman JR Henao-Tamayo MI Agger EM. The memory immune response to tuberculosis. Microbiol Spectr. (2016) 4:1–20. doi: 10.1128/microbiolspec.TBTB2-0009-2016 28087940

[13] Liu X Li H Li S Yuan J Pang Y. Maintenance and recall of memory T cell populations against tuberculosis: implications for vaccine design. Front Immunol. (2023) 14:1100741. doi: 10.3389/fimmu.2023.1100741 37063832 PMC10102482

[14] Vogelzang A Perdomo C Zedler U Kuhlmann S Hurwitz R Gengenbacher M. Central memory cd4+ T cells are responsible for the recombinant bacillus calmette-guerin deltaurec::Hly vaccine’s superior protection against tuberculosis. J Infect Dis. (2014) 210:1928–37. doi: 10.1093/infdis/jiu347 PMC4241943 24943726

[15] Tsai CY Oo M Peh JH Yeo BCM Aptekmann A Lee B. Splenic marginal zone B cells restrict mycobacterium tuberculosis infection by shaping the cytokine pattern and cell-mediated immunity. Cell Rep. (2024) 43:114426. doi: 10.1016/j.celrep.2024.114426 38959109 PMC11307145

[16] Nieuwenhuizen NE Kaufmann SHE. Next-generation vaccines based on bacille calmette-guerin. Front Immunol. (2018) 9:121. doi: 10.3389/fimmu.2018.00121 29459859 PMC5807593

[17] Mackay F Schneider P Rennert P Browning J. Baff and april: A tutorial on B cell survival. Annu Rev Immunol. (2003) 21:231–64. doi: 10.1146/annurev.immunol.21.120601.141152 12427767

[18] Moisini I Davidson A. Baff: A local and systemic target in autoimmune diseases. Clin Exp Immunol. (2009) 158:155–63. doi: 10.1111/j.1365-2249.2009.04007.x PMC2768805 19737141

[19] Baert L Manfroi B Casez O Sturm N Huard B. The role of april - a proliferation inducing ligand - in autoimmune diseases and expectations from its targeting. J Autoimmun. (2018) 95:179–90. doi: 10.1016/j.jaut.2018.10.016 30385081

[20] Cerutti A Cols M Puga I. Marginal zone B cells: virtues of innate-like antibody-producing lymphocytes. Nat Rev Immunol. (2013) 13:118–32. doi: 10.1038/nri3383 PMC3652659 23348416

[21] Mahairas GG Sabo PJ Hickey MJ Singh DC Stover CK. Molecular analysis of genetic differences between mycobacterium bovis bcg and virulent M. Bovis J Bacteriol. (1996) 178:1274–82. doi: 10.1128/jb.178.5.1274-1282.1996 PMC177799 8631702

[22] Brosch R Gordon SV Billault A Garnier T Eiglmeier K Soravito C. Use of a mycobacterium tuberculosis H37rv bacterial artificial chromosome library for genome mapping, sequencing, and comparative genomics. Infect Immun. (1998) 66:2221–9. doi: 10.1128/IAI.66.5.2221-2229.1998 PMC108185 9573111

[23] Pillai S Cariappa A. The follicular versus marginal zone B lymphocyte cell fate decision. Nat Rev Immunol. (2009) 9:767–77. doi: 10.1038/nri2656 19855403

[24] Gengenbacher M Vogelzang A Schuerer S Lazar D Kaiser P Kaufmann SH. Dietary pyridoxine controls efficacy of vitamin B6-auxotrophic tuberculosis vaccine bacillus calmette-guerin deltaurec::Hly deltapdx1 in mice. mBio. (2014) 5:e01262–14. doi: 10.1128/mBio.01262-14 PMC4049106 24895310

[25] Khatri B Keeble J Dagg B Kaveh DA Hogarth PJ Ho MM. Efficacy and immunogenicity of different bcg doses in balb/C and cb6f1 mice when challenged with H37rv or Beijing hn878. Sci Rep. (2021) 11:23308. doi: 10.1038/s41598-021-02442-5 34857776 PMC8639814

[26] Lalor MK Floyd S Gorak-Stolinska P Ben-Smith A Weir RE Smith SG. Bcg vaccination induces different cytokine profiles following infant bcg vaccination in the uk and Malawi. J Infect Dis. (2011) 204:1075–85. doi: 10.1093/infdis/jir515 PMC3164434 21881123

[27] Djoba Siawaya JF Beyers N van Helden P Walzl G. Differential cytokine secretion and early treatment response in patients with pulmonary tuberculosis. Clin Exp Immunol. (2009) 156:69–77. doi: 10.1111/j.1365-2249.2009.03875.x 19196252 PMC2673743

[28] Choong ML Yong YP Tan AC Luo B Lodish HF. Lix: A chemokine with a role in hematopoietic stem cells maintenance. Cytokine. (2004) 25:239–45. doi: 10.1016/j.cyto.2003.11.002 15036238

[29] Shi Y Liu CH Roberts AI Das J Xu G Ren G. Granulocyte-macrophage colony-stimulating factor (Gm-csf) and T-cell responses: what we do and don’t know. Cell Res. (2006) 16:126–33. doi: 10.1038/sj.cr.7310017 16474424

[30] Loetscher M Gerber B Loetscher P Jones SA Piali L Clark-Lewis I. Chemokine receptor specific for ip10 and mig: structure, function, and expression in activated T-lymphocytes. J Exp Med. (1996) 184:963–9. doi: 10.1084/jem.184.3.963 PMC2192763 9064356

[31] Millington KA Innes JA Hackforth S Hinks TS Deeks JJ Dosanjh DP. Dynamic relationship between ifn-gamma and il-2 profile of mycobacterium tuberculosis-specific T cells and antigen load. J Immunol. (2007) 178:5217–26. doi: 10.4049/jimmunol.178.8.5217 PMC2743164 17404305

[32] Gengenbacher M Nieuwenhuizen N Vogelzang A Liu H Kaiser P Schuerer S. Deletion of nuog from the vaccine candidate mycobacterium bovis bcg deltaurec::Hly improves protection against tuberculosis. mBio. (2016) 7:1–10. doi: 10.1128/mBio.00679-16 PMC4895111 27222470

[33] Rao M Vogelzang A Kaiser P Schuerer S Kaufmann SH Gengenbacher M. The tuberculosis vaccine candidate bacillus calmette-guerin deltaurec::Hly coexpressing human interleukin-7 or -18 enhances antigen-specific T cell responses in mice. PloS One. (2013) 8:e78966. doi: 10.1371/journal.pone.0078966 24236077 PMC3827306

[34] Butkeviciute E Jones CE Smith SG. Heterologous effects of infant bcg vaccination: potential mechanisms of immunity. Future Microbiol. (2018) 13:1193–208. doi: 10.2217/fmb-2018-0026 PMC6190278 30117744

[35] Rakshit S Adiga V Ahmed A Parthiban C Chetan Kumar N Dwarkanath P. Evidence for the heterologous benefits of prior bcg vaccination on covishield vaccine-induced immune responses in sars-cov-2 seronegative young Indian adults. Front Immunol. (2022) 13:985938. doi: 10.3389/fimmu.2022.985938 36268023 PMC9577398

[36] Ylosmaki E Fusciello M Martins B Feola S Hamdan F Chiaro J. Novel personalized cancer vaccine platform based on bacillus calmette-guerin. J Immunother Cancer. (2021) 9:1–13. doi: 10.1136/jitc-2021-002707 PMC8286790 34266884

[37] Smulski CR Eibel H. Baff and baff-receptor in B cell selection and survival. Front Immunol. (2018) 9:2285. doi: 10.3389/fimmu.2018.02285 30349534 PMC6186824

[38] Liu Z Davidson A. Baff and selection of autoreactive B cells. Trends Immunol. (2011) 32:388–94. doi: 10.1016/j.it.2011.06.004 PMC3151317 21752714

[39] Davidson A. Targeting baff in autoimmunity. Curr Opin Immunol. (2010) 22:732–9. doi: 10.1016/j.coi.2010.09.010 PMC2997938 20970975

[40] Novak AJ Darce JR Arendt BK Harder B Henderson K Kindsvogel W. Expression of bcma, taci, and baff-R in multiple myeloma: A mechanism for growth and survival. Blood. (2004) 103:689–94. doi: 10.1182/blood-2003-06-2043 14512299

[41] Pers JO Daridon C Devauchelle V Jousse S Saraux A Jamin C. Baff overexpression is associated with autoantibody production in autoimmune diseases. Ann N Y Acad Sci. (2005) 1050:34–9. doi: 10.1196/annals.1313.004 16014518

[42] Knight AK Radigan L Marron T Langs A Zhang L Cunningham-Rundles C. High serum levels of Baff, April, and Taci in common variable immunodeficiency. Clin Immunol. (2007) 124:182–9. doi: 10.1016/j.clim.2007.04.012 PMC2491330 17556024

[43] Gehad A Teague JE Matos TR Huang V Yang C Watanabe R. A primary role for human central memory cells in tissue immunosurveillance. Blood Adv. (2018) 2:292–8. doi: 10.1182/bloodadvances.2017011346 PMC5812324 29437556

[44] Sheridan BS Lefrancois L. Regional and mucosal memory T cells. Nat Immunol. (2011) 12:485–91. doi: 10.1038/ni.2029 PMC3224372 21739671

[45] Woodland DL Kohlmeier JE. Migration, maintenance and recall of memory T cells in peripheral tissues. Nat Rev Immunol. (2009) 9:153–61. doi: 10.1038/nri2496 19240755

[46] Ritz N Hanekom WA Robins-Browne R Britton WJ Curtis N. Influence of bcg vaccine strain on the immune response and protection against tuberculosis. FEMS Microbiol Rev. (2008) 32:821–41. doi: 10.1111/j.1574-6976.2008.00118.x 18616602

[47] Tanner R Villarreal-Ramos B Vordermeier HM McShane H. The humoral immune response to bcg vaccination. Front Immunol. (2019) 10:1317. doi: 10.3389/fimmu.2019.01317 31244856 PMC6579862

