# Supplementary material for: BAFF and APRIL immunotherapy following Bacille Calmette-Guérin vaccination enhances protection against pulmonary tuberculosis in mice
Source: Front Immunol. 2025 Feb 6;16:1551183. doi: 10.3389/fimmu.2025.1551183 (PMC11839638; doi:10.3389/fimmu.2025.1551183)
Supplement: Supplementary file 1 [file DataSheet1.pdf]

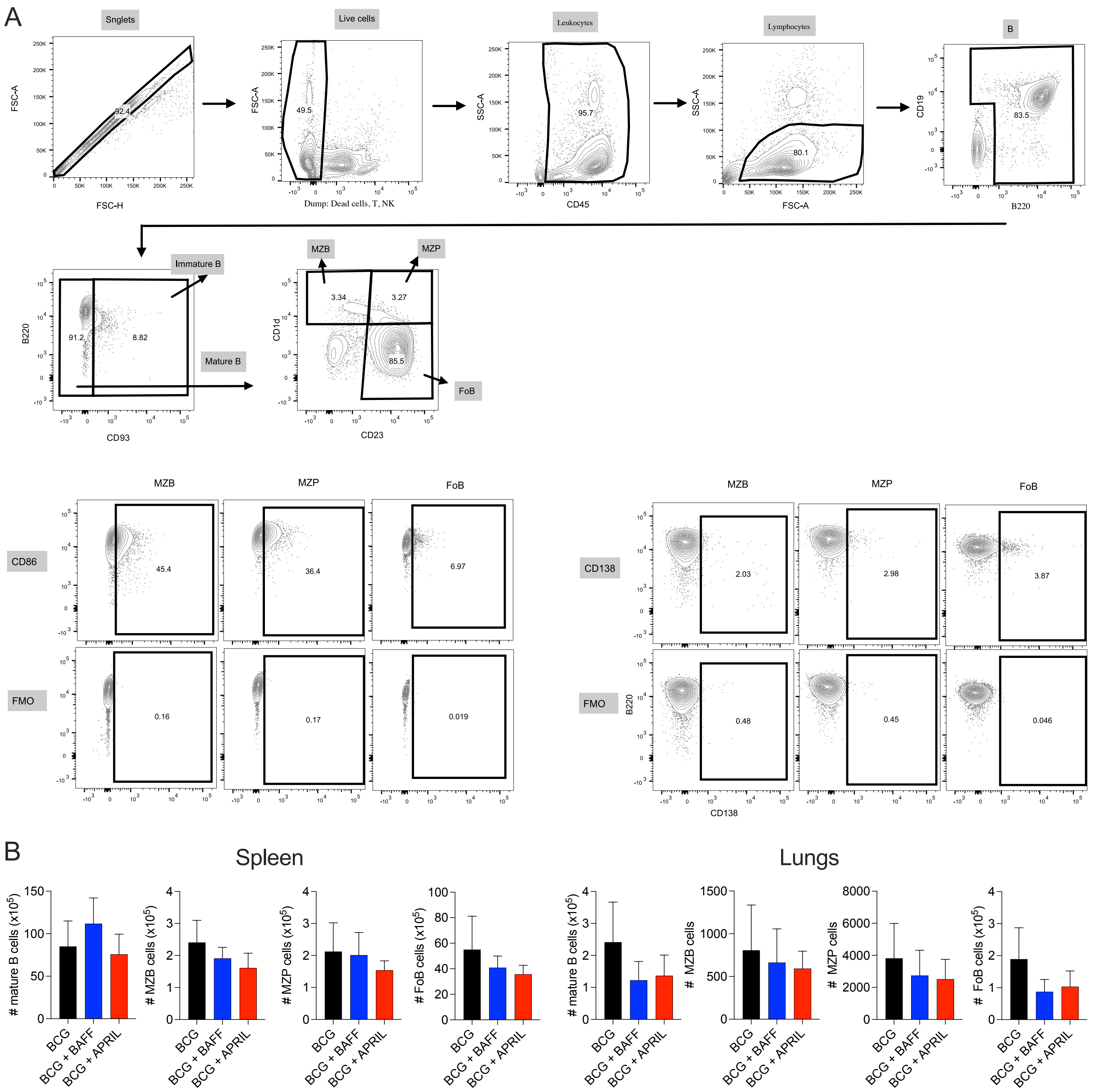

Supplementary Figure 1. Gating strategy for B lymphocytes and absolute numbers of B cell subsets. **(A)** Gating strategy for identification of B-cell subsets. Mature B cells (B220+ CD93+) were gated into three subsets: MZB ($CD1d^{hi}$ $CD23^{lo}$), FoB ($CD1d^{mi}$ $CD23^{hi}$) and MZP ($CD1d^{hi}$ $CD23^{hi}$). **(B)** Absolute numbers of all mature B cells, and MZB, MZP, and FoB cells in the spleen and in the lungs of BCG-vaccinated mice after immunotherapy with BAFF and APRIL (day 60, Figure 2A). Mean and SD are shown. Statistical analysis was performed by one-way ANOVA and multiple comparisons by Dunnett's posttest using the BCG-vaccinated group as a reference.

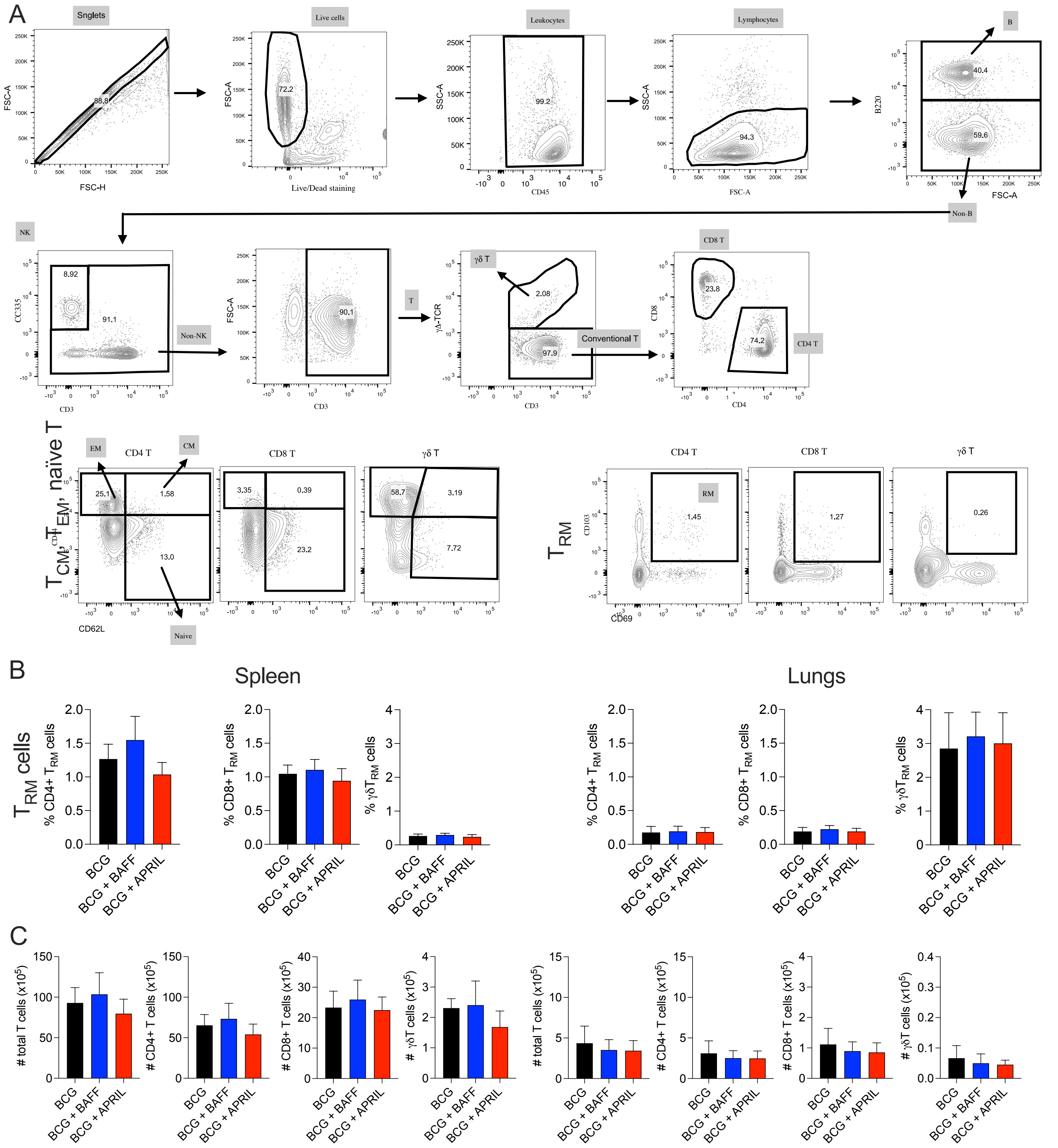

Supplementary Figure 2. Gating strategy for T cells and analysis of T cell subsets with tissue-resident-memory phenotype. **(A)** Gating strategy to identify CD4, CD8, and γδT and, and their central memory, effector memory and tissue-resident memory phenotypes. **(B)** Frequencies of tissue-resident memory CD4, CD8, and γδT cells in the spleen and the lungs of BCG-vaccinated mice after immunotherapy with BAFF and APRIL (day 60, Figure 2A). **(C)** Absolute numbers of all T cells, and CD4, CD8, and γδT cells in the spleen and the lungs of BCG-vaccinated mice after immunotherapy with BAFF and APRIL (day 60, Figure 2A). Mean and SD are shown. Statistical analysis was performed by one-way ANOVA and multiple comparisons by Dunnett's posttest using the BCG-vaccinated group as a reference.

Supplementary Table 1 Cytokine and chemokine concentrations in draining lymph nodes, spleen and lungs at 60 days post BCG vaccination

Lymph Nodes	BCG[#] (pg/mL)	BCG+BAFF[#] (pg/mL)	Fold Change	*p*-value	Significance	BCG+APRIL[#] (pg/mL)	Fold Change	*p*-value	Significance
Eotaxin	61.11±38.60	314.33±397.11	5.14	0.2639	ns	231.91±230.21	3.79	0.5117	ns
G-CSF	4407.02±2609.13	1872.41±957.30	-2.35	0.0595	ns	4769.83±780.02	1.08	0.9180	ns
GM-CSF	50.00±31.72	32.35±3.83	-1.55	0.2792	ns	36.53±7.99	-1.37	0.4500	ns
IFN-γ	29.07±31.56	4.00±1.01	-7.26	0.0898	ns	3.83±2.01	-7.59	0.0875	ns
IL-1α	579.31±274.22	324.67±80.75	-1.78	0.1092	ns	319.76±182.75	-1.81	0.1021	ns
IL-1β	66.62±46.17	34.03±18.91	-1.96	0.2235	ns	30.71±23.74	-2.17	0.1724	ns
IL-2	24.50±5.23	12.48±4.84	-1.96	0.0042	**	10.99±4.67	-2.23	0.0018	**
IL-3	< MinDC*	< MinDC*	NA	NA	NA	< MinDC*	NA	NA	NA
IL-4	2.40±1.93	8.36±6.99	3.49	0.2162	ns	12.57±6.86	5.24	0.0298	*
IL-5	< MinDC*	1.29±1.14	NA	NA	NA	54.35±93.38	NA	NA	NA
IL-6	48.92±50.79	15.37±9.66	-3.18	0.1884	ns	15.38±12.95	-3.18	0.1886	ns
IL-7	8.27±4.13	9.06±2.91	1.10	0.9028	ns	8.18±2.61	-1.01	0.9986	ns
IL-9	590.69±216.20	280.04±61.77	-2.11	0.0095	**	312.99±106.89	-1.89	0.0184	*
IL-10	10.56±3.07	8.21±4.49	-1.29	0.6035	ns	11.13±5.02	1.05	0.9674	ns
IL-12 (p40)	33.14±8.60	17.41±18.64	-1.90	0.2125	ns	25.54±16.21	-1.30	0.6510	ns
IL-12 (p70)	< MinDC*	< MinDC*	NA	NA	NA	< MinDC*	NA	NA	NA
IL-13	< MinDC*	< MinDC*	NA	NA	NA	< MinDC*	NA	NA	NA
IL-15	55.47±10.10	22.05±13.63	-2.52	0.0044	**	34.24±13.45	-1.62	0.0524	ns
IL-17	5.57±4.10	2.70±1.22	-2.06	0.1923	ns	2.86±1.64	-1.94	0.2233	ns
IP-10	253.76±224.40	504.01±263.58	1.99	0.1915	ns	285.75±200.16	1.13	0.9656	ns
KC	448.45±258.62	270.66±140.52	-1.66	0.3908	ns	405.20±265.34	-1.11	0.9372	ns
LIF	12.97±8.38	17.45±12.61	1.35	0.6618	ns	12.30±4.38	-1.05	0.9902	ns
LIX	< MinDC*	< MinDC*	NA	NA	NA	< MinDC*	NA	NA	NA
MCP-1	1548.18±1157.21	919.87±504.25	-1.68	0.4255	ns	992.57±769.44	-1.56	0.5034	ns
M-CSF	27.83±5.30	18.62±5.36	-1.49	0.0196	*	21.34±3.57	-1.30	0.0967	ns
MIG	4469.99±3519.50	3044.91±1209.52	-1.47	0.5274	ns	2375.81±1302.29	-1.88	0.2846	ns
MIP-1α	66.23±36.71	77.21±37.89	1.17	0.9602	ns	141.84±115.90	2.14	0.2198	ns
MIP-1β	219.71±31.98	189.36±113.33	-1.16	0.9541	ns	235.02±207.80	1.07	0.9879	ns
MIP-2	99.37±22.61	124.41±37.82	1.25	0.4074	ns	121.10±32.31	1.22	0.4605	ns
RANTES	679.55±259.89	347.18±126.32	-1.96	0.0775	ns	361.03±282.08	-1.88	0.0913	ns
TNF-α	61.08±39.75	37.72±15.01	-1.62	0.3631	ns	38.33±26.11	-1.59	0.3800	ns
VEGF	204.33±242.15	83.22±66.84	-2.46	0.3545	ns	80.49±44.10	-2.54	0.3403	ns

Spleen	BCG[#] (pg/mL)	BCG+BAFF[#] (pg/mL)	Fold Change	*p*-value	Significance	BCG+APRIL[#] (pg/mL)	Fold Change	*p*-value	Significance
Eotaxin	123.19±64.94	219.69±35.19	1.78	0.0268	*	223.29±56.24	1.81	0.0221	*
G-CSF	21.39±11.47	12.94±4.13	-1.65	0.1488	ns	10.60±2.95	-2.02	0.0626	ns
GM-CSF	7.57±6.52	38.76±12.50	4.99	0.0001	***	37.76±2.97	5.12	0.0002	***
IFN-γ	91.93±56.82	39.46±5.32	-2.33	0.0725	ns	49.90±25.90	-1.84	0.1572	ns
IL-1α	272.18±80.82	333.72±22.72	1.23	0.1475	ns	319.35±32.03	1.17	0.2918	ns
IL-1β	59.43±21.45	71.67±8.72	1.21	0.3579	ns	61.34±11.63	1.03	0.9706	ns
IL-2	20.60±6.84	5.47±0.96	-3.77	0.0044	**	11.25±8.31	-1.83	0.0632	ns
IL-3	< MinDC*	< MinDC*	NA	NA	NA	< MinDC*	NA	NA	NA
IL-4	1.48±0.30	1.96±0.24	1.32	0.2110	ns	3.47±0.69	2.35	<0.0001	****
IL-5	< MinDC*	< MinDC*	NA	NA	NA	< MinDC*	NA	NA	NA
IL-6	16.81±8.34	19.25±9.01	1.15	0.8236	ns	18.85±3.43	1.12	0.8712	ns
IL-7	7.24±2.78	8.15±1.22	1.13	0.7333	ns	9.32±2.09	1.29	0.2602	ns
IL-9	310.43±51.56	247.46±46.99	-1.25	0.0981	ns	216.73±41.96	-1.43	0.0154	*
IL-10	21.61±6.91	17.98±5.31	-1.20	0.5895	ns	20.53±6.96	-1.05	0.9506	ns
IL-12 (p40)	16.35±1.88	< MinDC*	NA	NA	NA	< MinDC*	NA	NA	NA
IL-12 (p70)	< MinDC*	< MinDC*	NA	NA	NA	< MinDC*	NA	NA	NA
IL-13	< MinDC*	< MinDC*	NA	NA	NA	< MinDC*	NA	NA	NA
IL-15	50.89±7.47	28.14±4.30	-1.81	0.0517	ns	46.28±23.56	-1.10	0.8357	ns
IL-17	5.13±2.36	3.57±0.96	-1.44	0.2123	ns	4.66±0.52	-1.10	0.8414	ns
IP-10	925.56±335.62	1246.80±75.30	1.35	0.0658	ns	1148.39±148.59	1.24	0.2188	ns
KC	238.66±50.97	222.28±93.07	-1.07	0.9032	ns	201.38±54.47	-1.19	0.6122	ns
LIF	< MinDC*	< MinDC*	NA	NA	NA	< MinDC*	NA	NA	NA
LIX	1384.40±485.56	3326.25±575.13	2.40	<0.0001	****	2753.19±323.20	1.99	0.0012	**
MCP-1	183.96±95.24	137.53±16.45	-1.34	0.3636	ns	128.54±22.26	-1.43	0.2544	ns
M-CSF	106.11±60.72	22.63±7.20	-4.69	0.0060	**	23.73±11.99	-4.47	0.0065	**
MIG	1006.23±373.38	4302.16±250.69	4.28	0.0004	***	4801.54±1670.54	4.77	0.0001	***
MIP-1α	151.57±45.52	199.80±27.68	1.32	0.0572	ns	189.89±11.08	1.25	0.1351	ns
MIP-1β	621.93±241.79	989.63±149.41	1.59	0.0086	**	938.49±57.25	1.51	0.0207	*
MIP-2	103.87±23.91	112.00±28.79	1.08	0.9265	ns	116.17±57.44	1.12	0.8424	ns
RANTES	972.31±528.24	875.80±60.87	-1.11	0.8570	ns	849.95±199.92	-1.14	0.7837	ns
TNF-α	18.67±4.01	14.11±1.03	-1.32	0.0278	*	12.59±1.55	-1.48	0.0050	**
VEGF	18.53±18.62	2.50±0.43	-7.41	0.0649	ns	2.86±0.63	-6.48	0.0713	ns

Lungs	BCG# (pg/mL)	BCG+BAFF# (pg/mL)	Fold Change	*p*-value	Significance	BCG+APRIL# (pg/mL)	Fold Change	*p*-value	Significance
Eotaxin	1656.54±528.47	778.49±223.39	-2.13	0.0027	**	591.95±107.05	-2.80	0.0006	***
G-CSF	31.80±11.51	31.49±13.49	-1.01	0.9983	ns	18.13±2.51	-1.75	0.1027	ns
GM-CSF	21.38±11.61	34.87±9.73	1.63	0.0873	ns	36.06±7.49	1.68	0.0623	ns
IFN-γ	77.48±12.07	17.05±14.11	-4.55	<0.0001	****	7.13±7.82	-10.87	<0.0001	****
IL-1α	384.53±77.18	180.74±41.34	-2.13	0.0004	***	154.17±48.47	-2.49	0.0001	***
IL-1β	14.98±5.54	11.77±4.29	-1.27	0.6398	ns	11.67±8.24	-1.28	0.6242	ns
IL-2	51.85±17.96	10.43±2.98	-4.97	0.0001	***	12.89±6.37	-4.02	0.0002	***
IL-3	< MinDC*	< MinDC*	NA	NA	NA	< MinDC*	NA	NA	NA
IL-4	< MinDC*	< MinDC*	NA	NA	NA	< MinDC*	NA	NA	NA
IL-5	13.32±10.88	3.31±1.71	-4.02	0.3542	ns	10.46±14.94	-1.27	0.9028	ns
IL-6	130.05±17.00	31.35±15.37	-4.15	<0.0001	****	16.26±16.77	-8.00	<0.0001	****
IL-7	7.89±2.67	7.05±2.75	-1.12	0.8702	ns	7.50±3.53	-1.05	0.9697	ns
IL-9	247.34±48.59	179.25±30.73	-1.38	0.1859	ns	260.70±90.97	1.05	0.9199	ns
IL-10	19.41±2.93	7.51±3.30	-2.58	0.0002	***	12.04±3.82	-1.61	0.0088	**
IL-12 (p40)	25.65±4.54	19.63±3.26	-1.31	0.3501	ns	14.06±9.01	-1.82	0.0418	*
IL-12 (p70)	< MinDC*	< MinDC*	NA	NA	NA	< MinDC*	NA	NA	NA
IL-13	< MinDC*	< MinDC*	NA	NA	NA	< MinDC*	NA	NA	NA
IL-15	60.91±13.48	34.78±20.58	-1.75	0.0485	*	45.08±14.20	-1.35	0.2557	ns
IL-17	5.72±5.85	1.41±0.60	-4.06	0.2452	ns	3.47±4.79	-1.65	0.6405	ns
IP-10	467.34±208.62	435.85±194.82	-1.07	0.9363	ns	279.28±21.78	-1.67	0.1671	ns
KC	596.20±120.49	270.54±138.54	-2.20	0.0012	**	210.54±66.28	-2.83	0.0003	***
LIF	< MinDC*	< MinDC*	NA	NA	NA	< MinDC*	NA	NA	NA
LIX	2702.76±1789.08	2629.03±2381.02	-1.03	0.9972	ns	1985.95±1317.16	-1.36	0.7756	ns
MCP-1	979.89±244.93	770.38±199.11	-1.27	0.2004	ns	586.37±127.72	-1.67	0.0150	*
M-CSF	62.29±8.87	51.62±8.12	-1.21	0.1118	ns	39.66±7.76	-1.57	0.0018	**
MIG	154.55±50.00	125.55±45.61	-1.23	0.4808	ns	103.56±30.97	-1.49	0.1473	ns
MIP-1α	73.90±15.52	81.92±14.06	1.11	0.5023	ns	68.29±3.81	-1.08	0.6990	ns
MIP-1β	196.87±72.83	260.93±61.48	1.33	0.1674	ns	228.72±20.95	1.16	0.5877	ns
MIP-2	137.11±21.78	163.22±30.62	1.19	0.2899	ns	177.72±32.37	1.30	0.0791	ns
RANTES	222.44±93.98	196.48±30.31	-1.13	0.7396	ns	189.02±43.10	-1.18	0.6164	ns
TNF-α	6.42±0.97	6.45±2.41	1.00	0.9998	ns	6.17±2.65	-1.04	0.9750	ns
VEGF	216.26±46.31	117.59±30.26	-1.84	0.0021	**	96.66±30.88	-2.24	0.0004	***

*MinDC (Minimum Detectable Concentration). MinDC values for each cytokine were provided by the manufacturer. #Mean and standard deviation.
